# The mediating role of synovitis in meniscus pathology and knee osteoarthritis radiographic progression

**DOI:** 10.1038/s41598-024-63291-6

**Published:** 2024-05-29

**Authors:** Hui Deng, Zhijun Chen, Jiawei Kang, Jun Liu, Shenliang Chen, Mingzhang Li, Jun Tao

**Affiliations:** Department of Orthopedics, Second Affiliated Hospital of Nanchang University, Nanchang, 330006 China

**Keywords:** Meniscus extrusion, Meniscus tear, Synovitis, Mediation analysis, Knee osteoarthritis progression, Diseases, Medical research, Pathogenesis, Rheumatology

## Abstract

Meniscus pathologies (damage, extrusion) and synovitis are associated with knee osteoarthritis (KOA); however, whether synovitis mediates the relationship between meniscus pathologies and KOA radiographic progression remains unclear. We conducted an observational study in the Osteoarthritis Initiative (OAI) cohort, with a 48-month follow-up. Meniscus pathology and synovitis were measured by MRI osteoarthritis knee score (MOAKS) at baseline and 24 months, and a comprehensive synovitis score was calculated using effusion and Hoffa synovitis scores. The knee osteoarthritis radiographic progression was considered that Kellgren–Lawrence (KL) grade and joint space narrowing (JSN) grade at 48 months were increased compared to those at baseline. This study included a total of 589 participants, with KL grades mainly being KL1 (26.5%), KL2 (34.1%), and KL3 (30.2%) at baseline, while JSN grades were mostly 0 at baseline. A logistic regression model was used to analyze the relationship between meniscus pathology, synovitis, and KOA progression. Mediation analysis was used to evaluate the mediation effect of synovitis. The average age of the participants was 61 years old, 62% of which were female. The medial meniscus extrusion was longitudinally correlated with the progression of KL (odds ratio [OR]: 2.271, 95% confidence interval [CI]: 1.412–3.694) and medial JSN (OR: 3.211, 95% CI: 2.040–5.054). Additionally, the longitudinal correlation between medial meniscus damage and progression of KOA (OR: 1.853, 95% CI: 1.177–2.941) and medial JSN (OR: 1.655, 95% CI: 1.053–2.602) was significant. Synovitis was found to mediate the relationship between medial meniscus extrusion and KL and medial JSN progression at baseline (β: 0.029, 95% CI: 0.010–0.053; β: 0.022, 95% CI: 0.005–0.046) and beyond 24 months (β: 0.039, 95% CI: 0.016–0.068; β: 0.047, 95% CI: 0.020–0.078). However, we did not find evidence of synovitis mediating the relationship between meniscal damage and KOA progression. Synovitis mediates the relationship between medial meniscus extrusion (rather than meniscus damage) and KOA progression.

## Introduction

Osteoarthritis is a chronic degenerative disease that affects joints and surrounding tissues, with primary characteristics including articular cartilage injury, subchondral osteophyte formation, and peripheral synovitis^1^. The clinical manifestations of knee osteoarthritis (KOA) include joint pain and disability^2,3^. However, there is still no effective treatment to prevent or slow the progress of KOA.

Common risk factors of KOA include aging, female, genetic susceptibility, obesity, and joint related structural abnormalities^4^. An increasing body of evidence shows that meniscus extrusion, meniscus damage, and synovitis are all factors associated with KOA progression. The incidence of radiographic osteoarthritis (ROA) in knee joints with meniscus extrusion is three times higher than that in knee joints without extrusion^5^. Meniscus extrusion (especially medial) also accelerates the loss of knee cartilage, causes the progression of ROA, and increases the risk of total knee arthroplasty^6–8^. Sihvonen et al. found that most patients with meniscus damage experienced progression of tibia and femur KOA in a 5-year follow-up study^9^. In addition, synovitis can precede ROA and is associated with the risk of subsequent incidence of KOA^10^, in addition to being significantly associated with the risk of KOA progression^11^. Taken together, these studies suggest that patients with meniscus extrusion, meniscus damage, or synovitis may be at high risk of KOA acceleration.

The knee synovitis phenotype exists in both early and late KOA, but the specific cause of synovitis remains unclear^12^. Grainger et al. reported the association between medial meniscus extrusion and local synovitis, although this association was less obvious in the lateral compartment^13^. Interestingly, Favero et al. recently reported that when synovium of early osteoarthritis was cocultured with meniscus, the protein levels of matrix metalloproteinase-3 (MMP-3) and MMP-10 were increased, suggesting an intrinsic interaction between meniscus and synovitis^14^. However, it is unclear whether these meniscus pathologies cause the progression of KOA by influencing synovitis.

In this study, we evaluated the potential mediating role of synovitis between the progression of KOA and meniscus pathology. The main outcome of the survey was the progression of Kellgren–Lawrence (KL) grade and joint space narrowing (JSN), which are the most commonly used imaging features to characterize the progress of KOA^15^. We analyzed the extent to which synovitis mediated the causal relationship between meniscus pathology and KOA radiographic progression through the mediation model, to better understand and explain the functional pathways acting between them.

## Materials and methods

### Database and participants

The data were obtained from the Osteoarthritis Initiative (OAI) cohort (https://nda.nih.gov/oai), a multicenter observational cohort study initiated by the National Institutes of Health. Briefly, the study included clinical assessment data, X-ray data, MRI data, and a biospecimen repository of 4796 participants aged 45–79 years at baseline, whose study principles and general inclusion criteria have been previously described^16^. The OAI conducts one follow-up per year on participants, with a follow-up rate of more than 90% in the initial 48 months. The study was approved by the local institutional review board, all research was performed in accordance with relevant guidelines/regulations and with the Declaration of Helsinki, and all selected participants have provided informed consent (see https://oai.epi-ucsf.org)^17^.

The demographic data at baseline included the sex, age, race, and body mass index (BMI) of the participants. A participant’s knee injury history was defined as whether their knee had been seriously injured to limit their walking ability for at least 1 week previously. The KL grade for the knee and JSN score were evaluated from fixed flexion weight-bearing X-ray, and the radiographs were read centrally at Boston University^18^. Four clinical sites acquired MRIs of the knee using a 3 T MRI system (Trio; Siemens Healthcare). The meniscus extrusion was determined with Dual Echo Steady State (DESS) and two-dimensional weighted turbo spin-echo (2D-TSE) with sagittal slices. Hoffa synovitis was identified with axial fat-suppressed (FS) turbo spin-echo (TSE) images in both sagittal and coronal planes, while effusion synovitis was determined with axial multiplanar reformations (MPR) of the DESS sequence; these images were assessed through the semi-quantitative MRI osteoarthritis knee scoring system (MOAKS). In the absence of knowledge of the clinical characteristics of all participants, MRI images were read sequentially by musculoskeletal radiologists with extensive experience^19^. The readings from the OAI database and the reading projects IDs are 22 and 65. Project 22 visited 600 participants at baseline, 12 months, and 24 months. Prior to transferring images to Boston Imaging Core Lab (BICL) and the start of taking measurements, the MR images were blinded to the OAI Release ID. The images were assessed paired and with known chronological order under the supervision of Dr. Ali Guermazi. Cartilage morphology, bone marrow lesions (BMLs), osteophytes, meniscal damage, synovitis and effusion, and extra articular features such as cysts and bursitis were scored^20^. Project 65 visited 1033 participants at baseline, 12 months, 24 months, 36 months, and 48 months. This project was performed under the supervision of Dr. Kent Kwoh from the Arthritis Research Center at the University of Arizona (previously at the University of Pittsburgh Medical Center—one of the OAI Clinical Centers). Images were selected for reading by Dr. Kwoh and his team and were prepared and sent to BICL for readings which were done blinded to case/control status in the study. The images across all visits were assessed together, and with known chronological order, under the supervision of Dr. Ali Guermazi^21,22^.

**Figure 1:**
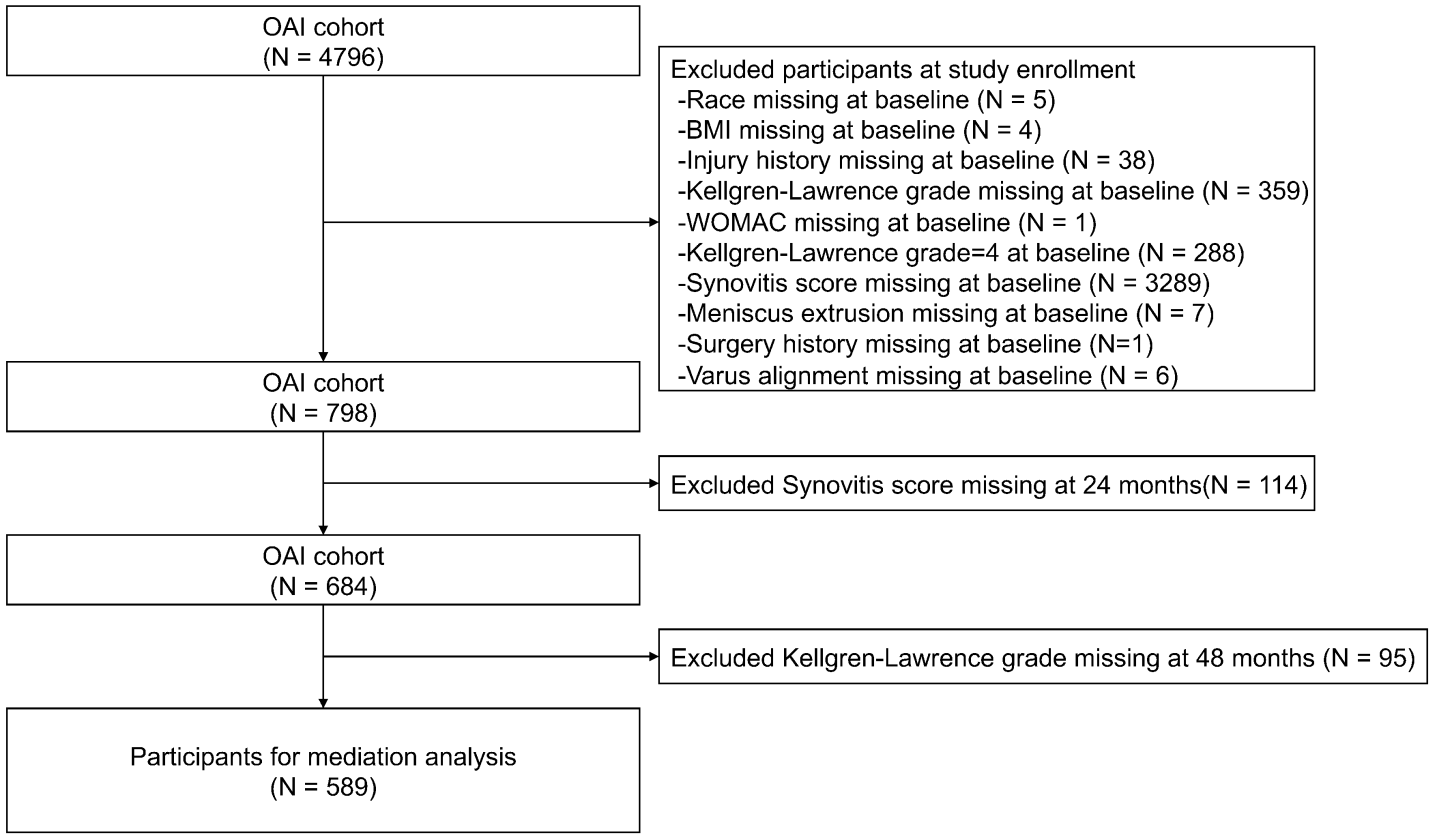
Participant selection from OAI cohort.

According to the algorithm used in previous OAI studies, a knee with a higher KL grade was targeted for inclusion in the analysis^23^. In cases of whether the radiographic severity was equal between the two knees, the knee with the higher Western Ontario and McMaster University Osteoarthritis Index (WOMAC) score was chosen. If the pain scores were equal between the two knees, the dominant knee was chosen. After excluding participants with missing demographic data at baseline (9 cases), missing Injury history (38 cases), missing KL grade or KL4 (647 cases), missing WOMAC scores (1 case), missing surgical history (1 case), missing Varus alignment assessment (6 cases), missing MOAKS scores for Synovitis (3289 cases), and missing MOAKS scores for Meniscus extrusion, there were 798 participants with complete records at baseline. At the second-year follow-up, after excluding 114 participants with missing MOAKS scores for Synovitis, there were 684 participants with complete records. At the fourth-year follow-up, after excluding 95 participants with missing KL grade, there were a total of 589 participants with complete records (Fig. 1).

### Meniscus pathology

The degree of meniscal pathology was scored using the MOAKS scoring system in the coronal plane. The meniscal extrusion was graded as follows: none, < 2 mm, 2.0–2.9 mm, 3.0–4.9 mm, or > 5 mm extrusion^24^. The meniscus morphology scores include 0 (normal meniscus), 1 (signal abnormality), 2 (radial tear), 3 (horizontal tear), 4 (vertical tear), 5 (complex tear), 6 (partial maceration), 7 (progressive partial maceration), and 8 (complete maceration). The intra-observer agreement for medial and lateral meniscal extrusion was 82% (95% confidence interval [CI]: 0.67–0.98) and 89% (95% CI: 0.75–1.00), respectively, for repeat measurements of meniscal extrusion. The inter-observer agreement for medial and lateral meniscal extrusion was 66% (95% CI: 0.46–0.86) and 79% (95% CI: 0.67–0.91), respectively. The intra-observer agreement for medial and lateral meniscal damage was 100% (95% CI: 1.00–1.00) and 91% (95% CI: 0.77–1.00), respectively, for repeat measurements of meniscal damage. The inter-observer agreement for medial and lateral meniscal damage was 97% (95% CI: 0.89–1.00) and 95% (95% CI: 0.58–1.00), respectively^19^. As mentioned earlier, if the meniscus extruded more than 3 mm at any location within the same compartment, meniscal extrusion in that compartment was defined as present^25–27^. Meniscus damage was considered maceration or tear of the meniscus at any location within the same compartment^28^.

### Synovitis

Effusion-synovitis was reported as a high signal in the joint cavity on the three-dimensional double-echo steady-state water excitation (3D-DESSWE) sequence post venous injection of contrast agent, which was scored from 0 to 3 (0 = normal, 1 = fluid persistence in the popliteal space, 2 = mild bulging of the suprapatellar bursa, and 3 = evidence of joint capsule distension)^29^. Hoffa-synovitis was reported as a high signal in the fat pad inter-region on the T2 fat suppressed sequence with a score of 0–3 (0 = normal, 1 = mild, 2 = moderate, and 3 = severe)^30^. We noted a small variability in the intra- and inter-observer measurements of Effusion-synovitis and Hoffa-synovitis. Specifically, the intra-observer agreement for Effusion-synovitis and Hoffa-synovitis were 95% (95% CI: 0.61–1.00) and 68% (95% CI: 0.38–0.99), respectively. The inter-observer agreement of Effusion-synovitis and Hoffa-synovitis were 91% (95% CI: 0.57–1.00) and 68% (95% CI: 0.38–0.99), respectively^31^. The total synovitis score is calculated by adding the Hoffa and Effusion scores together^32^.

### Osteoarthritis radiographic progression

The radiographic follow-up data of the participants after 48 months were limited; therefore, we only observed the KOA radiographic progression within 48 months. The KL grades of radiographic KOA severity (0 = no osteoarthritis; 1 = suspicious joint space narrowing; 2 = definite osteophytes and possible joint space narrowing; 3 = multiple osteophytes, definite joint space narrowing, and mild sclerosis; 4 = large osteophytes, obvious joint space narrowing, severe sclerosis, and deformity) and the extent of medial joint space narrowing (0–3) were scored according to the Osteoarthritis Research Society International (OARSI) atlas^33,34^. The reliability of these radiographic readings was good, with intra-observer consistency of 71% (95% CI: 0.55–0.87)^34^. An increase of ≥ 1 grade in KL grade from baseline to 48 months was considered progression for radiographic KOA, while any increase in the JSN score of the medial or lateral joint from baseline to 48 months was considered a progression of joint space narrowing^35^.

### Statistical analysis

Demographic characteristics are represented by means, standard deviations (SDs), percentages, median, and interquartile range (IQR). The relationship between meniscal pathology/synovitis and KL/JSN progression was examined through binary logistic regression analysis. The outcomes of this analysis are presented as odds ratios (ORs) accompanied by a 95% confidence interval (CI).

The “bruceR” package (version 0.8.10) was used to analyze the mediation model. Model 4 of “bruceR” package was selected to calculate the natural indirect effect, natural direct effect, and total effect, with adjustments for age, race, BMI, varus alignment, respective compartment meniscal posterior root tear, knee injury, knee surgery and/or sex, baseline KL grade, meniscus damage, and meniscal extrusion; this simulation model was repeated for 1000 bootstraps. In this model, the total effect represents the total influence of meniscal pathology on KL/JSN progression. Furthermore, the natural indirect effect represents the population mean of causal mediating, while the natural direct effect represents the population mean of direct effects. The proportion of meniscal pathology attributed to synovitis as an effector of KOA radiographic progression was estimated by dividing the natural indirect effect by the total effect. All data analysis was realized by R software (version 4.2.1, R Foundation for Statistical Computing, Vienna, Austria).

### Ethics declarations

The OAI study was approved by the institutional review boards at each OAI clinical site and the coordinating center (University of California, San Francisco) and participants provided written informed consent. This study obtained approval from the local Galician Ethics Committee (Comité Autonómico de Ética da Investigación de Galicia) with registry code 2018/129, All research was performed in accordance with relevant guidelines/regulations and with the Declaration of Helsinki.

## Results

### Participants’ characteristics

The demographic characteristics of the participants in this study are shown in Table 1. The baseline mean age (SD) of all of the participants was 61.25 ± 8.7 years, among whom, 62.5% were female and 83.0% were White. The mean BMI (SD) at baseline was 29.4 ± 4.5 kg/m^2^, with 45.2% reporting a previous knee injury and 23.6% reporting a history of knee surgery. Among all participants, 26.8% had varus alignment and 2.9% had meniscal posterior root tear. A total of 47.5% of the participants had medial meniscus damage, while 14.6% had lateral meniscus damage. The proportion of KL grades 1 (26.5%), 2 (34.1%), and 3 (30.2%) at baseline were similar across all participants, and 33.1% of participants had an increase in knee KL grade from baseline to 48 months. The medial and lateral JSN mean scores (SD) at baseline were 0.87 (0.83) and 0.07 (0.32), respectively, and 34.1% of the participants had medial or lateral progression of joint space narrowing at 48 months. A total of 207 participants had a medial meniscal extrusion, and 24 participants had a lateral meniscal extrusion. Additionally, a total of 433 participants had synovitis at baseline, and the mean synovitis summary score at 24 months increased by 0.136 for all participants. A total of 152 participants had both medial meniscal extrusion and medial meniscal damage, while 17 participants had both lateral meniscal extrusion and lateral meniscal damage. Interestingly, regardless of medial or lateral sides, participants with both meniscal damage and meniscal extrusion had higher meniscus morphology scores on average compared to those with meniscal damage but without meniscal extrusion.

**Table 1:** The baseline demographic characteristics of the participants in the study.

	All (N = 589)
Age, mean (SD)	61.25 (8.7)
Female, n (%)	368 (62.5%)
Male, n (%)	221 (37.5%)
Race, n (%)	
White	489 (83.0%)
Others	100 (17.0%)
BMI (kg/m^2^), mean (SD)	29.4 (4.5)
Injury history, n (%)	266 (45.2%)
Surgery history, n (%)	139 (23.6%)
Varus alignment, n (%)	158 (26.8%)
Meniscal posterior root tear at baseline, n (%)	
Medial	16 (2.7%)
Lateral	1 (0.2%)
Meniscal damage at baseline, n (%)	
Medial	280 (47.5%)
Lateral	86 (14.6%)
Kellgren and Lawrence (K–L) grade at baseline, n (%)	
0	54 (9.2%)
1	156 (26.5%)
2	201 (34.1%)
3	178 (30.2%)
Joint space narrowing at baseline, n (%)	
Medial	
0	247 (41.9%)
1	174 (29.6%)
2	168 (28.5%)
Lateral	
0	557 (94.5%)
1	21 (3.6%)
2	11 (1.9%)
Meniscus extrusion at baseline, n (%)	
Medial	207 (35.1%)
Lateral	24 (4.1%)
Synovitis summary score at baseline, median (IQR)	
Range 0–6	1 (0, 2)

*BMI* body mass index, *SD* standard deviation, *IQR* interquartile range.

### Direct effect

In Table 2, after multivariate adjustments (sex, age, race, BMI, varus alignment, respective compartment meniscal posterior root tear, knee injury history, knee surgery history, and KL grade), baseline medial meniscus extrusion was longitudinally correlated with KL progression at 48 months (OR: 2.504, 95% CI: 1.568, 4.046), and also with medial JSN progression at 48 months (OR: 3.336, 95% CI: 2.129, 5.228). There was no association between baseline lateral meniscus extrusion and KL progression at 48 months, but there was an association with lateral JSN progression at 48 months (OR: 12.228, 95% CI: 3.309, 45.189). Meniscal damage has similar results, with a significant correlation between baseline medial meniscus damage and KL progression at 48 months (OR: 2.190, 95% CI: 1.414, 3.429), while baseline medial or lateral meniscus damage is significantly correlated with medial (OR: 2.091, 95% CI: 1.357, 3.223) or lateral (OR: 10.284, 95% CI: 3.804, 27.800) JSN progression at 48 months, respectively. At baseline, synovitis was also longitudinally associated with KL progression (OR: 1.425, 95% CI: 1.216, 1.677), medial JSN progression (OR: 1.329, 95% CI: 1.140, 1.548) or lateral JSN progression (OR: 1.747, 95% CI: 1.188, 2.568). The longitudinal association between synovitis at 24 months and KL progression (OR: 1.522, 95% CI: 1.306, 1.782) medial JSN progression (OR: 1.620, 95% CI: 1.387, 1.891) or lateral JSN progression (OR: 1.457, 95% CI: 1.023, 2.076) at 48 months is still significant; in model 2, these associations remained significant after additional adjustment for baseline meniscus damage (for meniscal extrusion) or meniscal extrusion (for meniscus damage).

**Table 2:** Associations of meniscus pathology and synovitis score with knee osteoarthritis radiographic progression. Significant values are in bold.

	Model 1^a^		Model 2^b^	
	OR (95% CI)	*P*-value	OR (95% CI)	*P*-value
KL progression				
Baseline				
Medial meniscus extrusion	2.504 (1.568, 4.046)	**< 0.01**	2.271 (1.412, 3.694)	**< 0.01**
Lateral meniscus extrusion	0.464 (0.129, 1.314)	0.18	0.397 (0.108, 1.156)	0.12
Medial meniscus damage	2.190 (1.414, 3.429)	**< 0.01**	1.853 (1.177, 2.941)	**< 0.01**
Lateral meniscus damage	1.050 (0.618, 1.752)	0.85	1.192 (0.686, 2.042)	0.53
Synovitis score^c^	1.425 (1.216, 1.677)	**< 0.01**	1.418 (1.208, 1.672)	**< 0.01**
Baseline to 24 months				
Synovitis score^c^	1.522 (1.306, 1.782)	**< 0.01**	1.511 (1.294, 1.772)	**< 0.01**
Medial JSN progression				
Baseline				
Medial meniscus extrusion	3.336 (2.129, 5.228)	**< 0.01**	3.211 (2.040, 5.054)	**< 0.01**
Medial meniscus damage	2.091 (1.357, 3.223)	**< 0.01**	1.655 (1.053, 2.602)	**0.03**
Synovitis score^c^	1.329 (1.140, 1.548)	**< 0.01**	1.325 (1.137, 1.545)	**< 0.01**
Baseline to 24 months				
Synovitis score^c^	1.620 (1.387, 1.891)	**< 0.01**	1.613 (1.381, 1.884)	**< 0.01**
Lateral JSN progression				
Baseline				
Lateral meniscus extrusion	12.228 (3.309, 45.189)	**< 0.01**	10.604 (2.797, 40.199)	**< 0.01**
Lateral meniscus damage	10.284 (3.804, 27.800)	**< 0.01**	7.543 (2.571, 22.130)	**< 0.01**
Synovitis score^c^	1.747 (1.188, 2.568)	**< 0.01**	1.691 (1.153, 2.481)	**< 0.01**
Baseline to 24 months				
Synovitis score^c^	1.457 (1.023, 2.076)	**0.04**	1.391 (0.973, 1.989)	**< 0.01**

^a^Adjusted for sex, age, race, body mass index, varus alignment, respective compartment meniscal posterior root tear, injury, surgery and Kellgren and Lawrence (KL) grade at baseline.

^b^Meniscal extrusion additionally adjusted for meniscal damage (tear or maceration), and meniscal damage additionally adjusted for meniscal extrusion.

^c^No adjusted for respective compartment meniscal posterior root tear.

### Indirect effect

**Figure 2:**
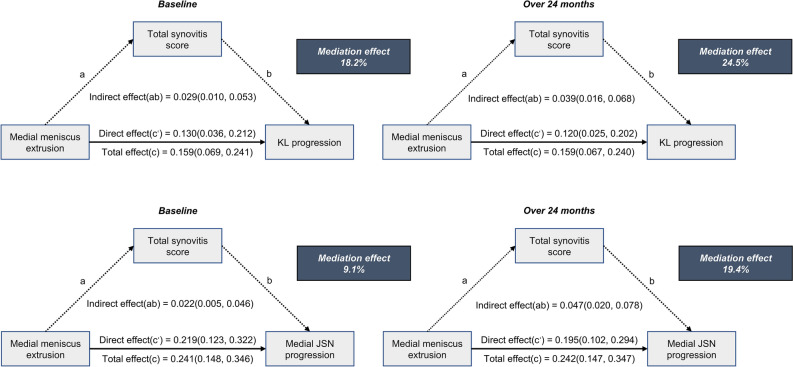
The mediation of synovitis in the relationship between medial meniscus extrusion and radiographic progression of knee osteoarthritis. Adjusted for sex, age, race, body mass index, varus alignment, respective compartment meniscal posterior root tear, meniscal damage (tear or maceration), injury, surgery, and Kellgren and Lawrence (KL) grade at baseline.

Given that both medial meniscus pathology and synovitis are associated with KL and JSN progression in the knee joint, we hypothesize that synovitis mediates the relationship between meniscus pathology and KOA radiographic progression at 48 months. In the mediation analysis, we included exposure factors (meniscus extrusion or damage), media (synovitis), results (KL progress or JSN progress), and potential confounding factors in the causal model for analysis (Fig. 2). Based on the mediation analysis, the impact of medial meniscus extrusion on knee KL progression was mediated by synovitis at baseline (0.029, 95% CI: 0.011, 0.054) and 24 months (0.040, 95% CI: 0.018, 0.069), respectively. Following further adjustment for meniscal damage (tear or maceration) in model 2, the aforementioned mediation ratio exhibited an increase. Specifically, the mediation ratio of synovitis at baseline was found to be 18.2% (Table 3), while at 24 months it rose to 24.5% (Table 4). However, we found no such mediating effect of synovitis at baseline or at 24 months on the association between meniscus damage and KL grade progression.

**Table 3:** Analysis of the association between meniscus pathology and Kellgren–Lawrence progression mediated by the total synovitis score at baseline. Significant values are in bold.

	Model 1^a^			Model 2^b^		
	*β*	95% CI	*P*-value	*β*	95% CI	*P*-value
Meniscus extrusion						
Medial						
Indirect effect	0.029	0.011, 0.054	**< 0.01**	0.029	0.010, 0.053	**< 0.01**
Direct effect	0.150	0.055, 0.231	**< 0.01**	0.130	0.036, 0.212	**< 0.01**
Total effect	0.179	0.092, 0.260	**< 0.01**	0.159	0.069, 0.241	**< 0.01**
Mediation	16.2%			18.2%		
Lateral						
Indirect effect	0.020	− 0.013, 0.060	0.29	0.018	− 0.013, 0.058	0.31
Direct effect	− 0.155	− 0.329, 0.019	0.09	− 0.173	− 0.329, − 0.003	**0.04**
Total effect	− 0.136	− 0.318, 0.045	0.15	− 0.155	− 0.320, 0.018	0.08
Mediation						
Meniscus damage						
Medial						
Indirect effect	− 0.001	− 0.016, 0.016	0.91	− 0.008	− 0.024, 0.006	0.26
Direct effect	0.152	0.071, 0.227	**< 0.01**	0.126	0.035, 0.202	**< 0.01**
Total effect	0.151	0.070, 0.226	**< 0.01**	0.117	0.031, 0.192	**< 0.01**
Mediation						
Lateral						
Indirect effect	0.023	0.004, 0.046	**0.04**	0.022	0.002, 0.045	0.06
Direct effect	− 0.022	− 0.118, 0.078	0.67	0.005	− 0.096, 0.108	0.93
Total effect	0.002	− 0.101, 0.105	0.98	0.027	− 0.077, 0.135	0.62
Mediation						

^a^Adjusted for sex, age, race, body mass index, varus alignment, respective compartment meniscal posterior root tear, injury, surgery and Kellgren and Lawrence (KL) grade at baseline.

^b^Meniscal extrusion additionally adjusted for meniscal damage (tear or maceration), and meniscal damage additionally adjusted for meniscal extrusion.

**Table 4:** Analysis of the association between meniscus pathology and Kellgren–Lawrence progression mediated by the total synovitis score over 24 months. Significant values are in bold.

	Model 1^a^			Model 2^b^		
	*β*	95% CI	*P*-value	*β*	95% CI	*P*-value
Meniscus extrusion						
Medial						
Indirect effect	0.040	0.018, 0.069	**< 0.01**	0.039	0.016, 0.068	**< 0.01**
Direct effect	0.139	0.044, 0.218	**< 0.01**	0.120	0.025, 0.202	**0.01**
Total effect	0.179	0.092, 0.262	**< 0.01**	0.159	0.067, 0.240	**< 0.01**
Mediation	22.3%			24.5%		
Lateral						
Indirect effect	0.001	− 0.034, 0.036	0.97	− 0.001	− 0.035, 0.034	0.08
Direct effect	− 0.134	− 0.318, 0.045	0.16	− 0.150	− 0.323, 0.026	0.09
Total effect	− 0.133	− 0.318, 0.046	0.15	− 0.151	− 0.320, 0.026	0.09
Mediation						
Meniscus damage						
Medial						
Indirect effect	0.009	− 0.009, 0.029	0.34	− 0.001	− 0.017, 0.016	0.93
Direct effect	0.141	0.060, 0.218	**< 0.01**	0.117	0.029, 0.194	**< 0.01**
Total effect	0.150	0.070, 0.227	**< 0.01**	0.116	0.029, 0.193	**< 0.01**
Mediation						
Lateral						
Indirect effect	0.020	− 0.002, 0.046	0.10	0.023	− 0.001, 0.049	0.08
Direct effect	− 0.018	− 0.119, 0.083	0.72	0.004	− 0.095, 0.106	0.94
Total effect	0.002	− 0.101, 0.105	0.97	0.026	− 0.076, 0.134	0.62
Mediation						

^a^Adjusted for sex, age, race, body mass index, varus alignment, respective compartment meniscal posterior root tear, injury, surgery and Kellgren and Lawrence (KL) grade at baseline.

^b^Meniscal extrusion additionally adjusted for meniscal damage (tear or maceration), and meniscal damage additionally adjusted for meniscal extrusion.

We also examined whether synovitis mediated the impact of meniscus extrusion on joint space narrowing progression. The results of the mediation analysis revealed a similar mediating effect. The effect of medial meniscus extrusion on the progression of medial joint space narrowing (JSN) at baseline (0.022, 95% CI: 0.005, 0.045) and 24 months (0.048, 95% CI: 0.021, 0.080) was found to be mediated by synovitis. This mediation ratio increased after accounting for meniscal damage (tear or maceration) in model 2, with mediation ratios of 9.1% (Table 5) and 19.4% (Table 6). Synovitis at baseline or 24 months did not mediate other association between meniscus damage and JSN progression. However, synovitis at 24 months mediated the association between concurrent meniscal damage and extrusion at baseline and KL progression (0.031, 95% CI: 0.009, 0.060) and medial JSN progression (0.038, 95% CI: 0.010, 0.072) at 48 months (Supplementary Table 1).

**Table 5:** Analysis of the association between meniscus pathology and joint space narrowing (JSN) progression mediated by the total synovitis score at baseline. Significant values are in bold.

	Model 1^a^			Model 2^b^		
	*β*	95% CI	*P*-value	*β*	95% CI	*P*-value
Meniscus extrusion						
Medial						
Indirect effect	0.022	0.005, 0.045	**0.03**	0.022	0.005, 0.046	**0.03**
Direct effect	0.227	0.127, 0.321	**< 0.01**	0.219	0.123, 0.322	**< 0.01**
Total effect	0.250	0.151, 0.348	**< 0.01**	0.241	0.148, 0.346	**< 0.01**
Mediation	8.8%			9.1%		
Lateral						
Indirect effect	0.012	− 0.009, 0.037	0.31	0.011	− 0.009, 0.036	0.35
Direct effect	0.157	0.013, 0.354	0.08	0.144	0.007, 0.336	0.09
Total effect	0.169	0.016, 0.367	0.06	0.155	0.012, 0.347	0.08
Mediation						
Meniscus damage						
Medial						
Indirect effect	− 0.001	− 0.013, 0.014	0.91	− 0.006	− 0.017, 0.005	0.27
Direct effect	0.147	0.055, 0.234	**< 0.01**	0.102	0.008, 0.193	**0.04**
Total effect	0.146	0.054, 0.233	**< 0.01**	0.096	0.001, 0.186	**0.05**
Mediation						
Lateral						
Indirect effect	0.007	− 0.000, 0.018	0.10	0.006	− 0.000, 0.015	0.14
Direct effect	0.098	0.034, 0.167	**< 0.01**	0.076	0.018, 0.143	**0.02**
Total effect	0.105	0.040, 0.173	**< 0.01**	0.082	0.023, 0.149	**0.01**
Mediation						

^a^Adjusted for sex, age, race, body mass index, varus alignment, respective compartment meniscal posterior root tear, injury, surgery and Kellgren and Lawrence (KL) grade at baseline.

^b^Meniscal extrusion additionally adjusted for meniscal damage (tear or maceration), and meniscal damage additionally adjusted for meniscal extrusion.

**Table 6:** Analysis of the association between meniscus pathology and joint space narrowing (JSN) progression mediated by the total synovitis score over 24 months. Significant values are in bold.

	Model 1^a^			Model 2^b^		
	*β*	95% CI	*P*-value	*β*	95% CI	*P*-value
Meniscal extrusion						
Medial						
Indirect effect	0.048	0.021, 0.080	**< 0.01**	0.047	0.020, 0.078	**< 0.01**
Direct effect	0.204	0.111, 0.297	**< 0.01**	0.195	0.102, 0.294	**< 0.01**
Total effect	0.251	0.151, 0.349	**< 0.01**	0.242	0.147, 0.347	**< 0.01**
Mediation	19.1%			19.4%		
Lateral						
Indirect effect	0.000	− 0.015, 0.016	0.97	− 0.000	− 0.015, 0.015	0.97
Direct effect	0.170	0.017, 0.366	0.06	0.158	0.016, 0.351	0.07
Total effect	0.170	0.018, 0.359	0.05	0.158	0.014, 0.348	0.07
Mediation						
Meniscus damage						
Medial						
Indirect effect	0.011	− 0.010, 0.035	0.35	− 0.001	− 0.020, 0.019	0.93
Direct effect	0.138	0.048, 0.220	**< 0.01**	0.098	0.006, 0.188	**0.03**
Total effect	0.148	0.056, 0.235	**< 0.01**	0.097	0.004, 0.187	**0.04**
Mediation						
Lateral						
Indirect effect	0.004	− 0.003, 0.012	0.27	0.004	− 0.002, 0.012	0.23
Direct effect	0.103	0.038, 0.174	**< 0.01**	0.079	0.023, 0.151	**0.02**
Total effect	0.107	0.042, 0.176	**< 0.01**	0.083	0.028, 0.154	**0.01**
Mediation						

^a^Adjusted for sex, age, race, body mass index, varus alignment, respective compartment meniscal posterior root tear, injury, surgery and Kellgren and Lawrence (KL) grade at baseline.

^b^Meniscal extrusion additionally adjusted for meniscal damage (tear or maceration), and meniscal damage additionally adjusted for meniscal extrusion.

## Discussion

In this large longitudinal observational study, we found that after adjusting for potential confounders, baseline medial meniscus pathology (extrusion and damage) was associated with KOA radiological progression over 4 years. Additionally, mediation analysis showed that synovitis at baseline mediated approximately one-fifth of the cases of medial meniscus extrusion and KOA radiological progression, while synovitis at 2 years mediated approximately one-quarter. These mediating models suggest that medial meniscus extrusion (instead of damage) affects synovitis and thus accelerates the progress of KOA.

The association between meniscus extrusion and KOA radiographic progression has been reported in the previous literature^7,36^. Our study adds to this evidence to prove the longitudinal association between medial meniscus extrusion and KOA progression. A previous study revealed that the narrowing of the initial joint space on conventional X-ray films was secondary to meniscus extrusion rather than thinning of articular cartilage^37^. Indeed, medial meniscus extrusion often occurs before cartilage injury, and changes in meniscus position account for a significant portion of minimum joint space width (mJSW) changes^7,38,39^. It is worth noting that the thickness of articular cartilage is also significantly correlated with the measurement of the joint space width of the medial lesion cavity^40^. Moreover, the pathological state of meniscus damage, including tear and maceration, has been reported as a progressive factor of KOA by several studies^41,42^. Our study further demonstrates that the medial meniscus is a strong risk factor for the progression of KOA. Therefore, the pathological status of the medial meniscus constitutes a public health problem in the aging population, and efforts need to be strengthened to better understand its etiology, prevention, and treatment.

As far as we know, this is the first study to examine synovitis as a mediator of meniscus pathology and KOA radiological progression. The value of mediation analysis in observational research has been recognized for its ability to quantitatively assess potential mechanisms^43^. Our results also have potential clinical significance. Some previous studies have focused on the causal relationship between meniscus extrusion and cartilage or joint space, while others have focused on the potential initial role of meniscus damage in the occurrence and progression of KOA^21^; however, it remains unclear whether the inflammatory phenotype mediates the correlation between the meniscus pathology and outcome of KOA. Our results suggest that the progression of KOA caused by medial meniscus extrusion (but not meniscal damage) is partly mediated by synovitis. There is increasing evidence that KOA radiology progression are closely related to the inflammatory phenotype^44,45^. Macrophages in synovial tissue are activated by related molecular patterns (DAMPs) such as cartilage fragments and aggrecans, secreting a large number of inflammatory factors and increasing the secretion of matrix metalloproteinases, thereby promoting the development of the inflammatory microenvironment and osteoarthritis^46^. Interestingly, several previous studies have evaluated the sagittal and coronal MR images of the knee joint and found that the extruded medial meniscus was related to intra-articular effusion^47,48^. Grainger et al. conducted a prospective study on 43 subjects and used gadolinium-enhanced MRI to evaluate synovitis. As a result, they found an association between medial meniscus extrusion and the severity of synovitis^13^. However, the small sample size represents a serious limitation of these conclusions. A recent study attempted to determine the molecules and pathways involved in meniscus-synovium interactions through co-culture experiments. The findings indicated that inflammatory molecules generated by the synovium and meniscus have the potential to initiate inflammatory signals in individuals with early osteoarthritis (OA), leading to the degradation of extracellular matrix during the pathological progression and advanced stages of OA^14^.

In terms of the potential mechanism, the change in knee joint load caused by meniscus extrusion may be the initial driving factor of the disease^5^. Atkinson, H.F et al. suggested that patients with KOA may exhibit mechanical inflammation and that the low load on the medial side of the knee may be positively associated with reduction in knee inflammation after high tibial osteotomy^49^. Compared to meniscus tearing, the reduction in meniscus coverage and height due to meniscus extrusion may lead to greater changes in tibial movement^50^. Meniscus extrusion of the knee joint can be regarded as a functional meniscus defect. Medial meniscus extrusion may increase the mechanical stress of articular cartilage and subchondral bone, thereby expediting the progression of synovitis. Conversely, synovitis may facilitate the deterioration of articular cartilage and subchondral bone by generating pro-inflammatory cytokines and matrix metalloproteinases^46^, ultimately contributing to the radiological advancement of knee osteoarthritis. Antony B et al. pointed out in their study that meniscal extrusion, rather than meniscal signal or tear, is associated with changes in knee joint structure, possibly due to the less destructive nature of these pathologies, while severe meniscal damage (maceration) is associated with knee joint pain and structural changes^51^. In our study, participants with severe meniscal damage at baseline were few, but still suggested an association between meniscal damage and radiographic progression of KOA. Our further investigation found that synovitis did not mediate this association. However, we cannot overlook the intra-articular inflammation caused by meniscal injury. Previous studies have found that synovitis frequently occurs in patients with traumatic meniscal injury who undergo arthroscopic meniscectomy and have no radiographic evidence of OA, and it is associated with increased pain and functional impairment^52^. Therefore, in some severe states of meniscal damage, especially traumatic meniscal damage caused by sports, we cannot ignore the role of synovitis in the progression of KOA, and more research is needed in the future to clarify this.

This study raises several key points that warrant attention. We conducted mediation analysis under the guidance of the AGReMA (Mediation Analysis Reporting Guidelines) statement to evaluate the causal pathway of the longitudinal mediation of synovitis in meniscus pathology and KOA radiology progression^43^. Additionally, the current study had advantages, in that this was a follow-up study based on a large-scale population, with a population design based on an OAI cohort. Nevertheless, this study still has several potential limitations. First, we selected the more serious knee joints in OAI for analysis, which may produce selection bias in the analysis results. However, to minimize the selection bias, we included as many samples as possible. We look forward to further research with larger sample size and more time points. Second, the synovitis and meniscus pathological scores of MOAKS were semi quantitative. However, the correlation between synovitis and histology assessed by MRI is relatively accurate and powerful^53^. Third, we used the comprehensive score of three positions of the meniscus as the score of two departments. The pathological state of the meniscus may affect different regions, although this requires further research to confirm. Finally, as this was an observational study, even if some potential confounding factors are adjusted as much as possible, there may be residual confounding effects.

## Conclusion

This longitudinal observational study provided evidence that baseline and 2-year synovitis mediated the relationship between medial meniscus extrusion rather than meniscus damage and radiological progression of KOA over 4 years. Our findings highlighted the importance of the complex interaction between medial meniscus extrusion, synovitis, and KOA progression. Further studies are required to confirm this finding.

### Supplementary Information

Supplementary Table 1.

## Data Availability

The datasets analyzed during this study are available from the Osteoarthritis Initiative website (https://nda.nih.gov/oai/).

## Abbreviations

KOA: Knee osteoarthritis
OAI: Osteoarthritis Initiative
MOAKS: MRI osteoarthritis knee score
KL: Kellgren–Lawrence
JSN: Joint space narrowing
ROA: Radiographic osteoarthritis
MMP-3: Matrix metalloproteinase-3
DESS: Dual Echo Steady State
2D-TSE: Two-dimensional weighted turbo spin-echo
FS: Fat-suppressed
TSE: Turbo spin-echo
MPR: Multiplanar reformations
WOMAC: McMaster University Osteoarthritis Index
3D-DESSWE: Three-dimensional double-echo steady-state water excitation
OARSI: Osteoarthritis Research Society International
SD: Standard deviations
IQR: Interquartile range
OR: Odds ratio
CI: Confidence interval
OA: Osteoarthritis
AGReMA: Mediation Analysis Reporting Guidelines

## References

[1] Hunter DJ, Bierma-Zeinstra S (2019). Osteoarthritis. Lancet.

[2] Nguyen U (2011). Increasing prevalence of knee pain and symptomatic knee osteoarthritis: Survey and cohort data. Ann. Intern. Med..

[3] Dillon CF, Rasch EK, Gu QP, Hirsch R (2006). Prevalence of knee osteoarthritis in the United States: Arthritis data from the Third National Health and Nutrition Examination Survey 1991–94. J. Rheumatol..

[4] Giorgino R (2023). Knee osteoarthritis: epidemiology, pathogenesis, and mesenchymal stem cells: What else is new? An update. Int. J. Mol. Sci..

[5] van der Voet JA (2023). Association between baseline meniscal extrusion and long-term incident knee osteoarthritis in two different cohorts. Semin. Arthritis Rheum..

[6] Raynauld J-P (2006). Long term evaluation of disease progression through the quantitative magnetic resonance imaging of symptomatic knee osteoarthritis patients: Correlation with clinical symptoms and radiographic changes. Arthritis Res. Ther..

[7] Teichtahl AJ (2017). Meniscal extrusion and bone marrow lesions are associated with incident and progressive knee osteoarthritis. Osteoarthr. Cartil..

[8] Gale DR (1999). Meniscal subluxation: Association with osteoarthritis and joint space narrowing. Osteoarthr. Cartil..

[9] Sihvonen R (2020). Arthroscopic partial meniscectomy for a degenerative meniscus tear: A 5 year follow-up of the placebo-surgery controlled FIDELITY (Finnish Degenerative Meniscus Lesion Study) trial. Br. J. Sports Med..

[10] Atukorala I (2016). Synovitis in knee osteoarthritis: A precursor of disease?. Ann. Rheum. Dis..

[11] Davis JE (2019). Effusion-synovitis and infrapatellar fat pad signal intensity alteration differentiate accelerated knee osteoarthritis. Rheumatology (Oxford).

[12] Sanchez-Lopez E, Coras R, Torres A, Lane NE, Guma M (2022). Synovial inflammation in osteoarthritis progression. Nat. Rev. Rheumatol..

[13] Grainger AJ, Rhodes LA, Keenan A-M, Emery P, Conaghan PG (2007). Quantifying peri-meniscal synovitis and its relationship to meniscal pathology in osteoarthritis of the knee. Eur. Radiol..

[14] Favero M (2019). Inflammatory molecules produced by meniscus and synovium in early and end-stage osteoarthritis: A coculture study. J. Cell Physiol..

[15] Joo PY, Borjali A, Chen AF, Muratoglu OK, Varadarajan KM (2022). Defining and predicting radiographic knee osteoarthritis progression: A systematic review of findings from the osteoarthritis initiative. Knee Surg. Sports Traumatol. Arthrosc..

[16] Deng H, Wu Y, Fan Z, Tang W, Tao J (2023). The association between patellofemoral grind and synovitis in knee osteoarthritis: Data from the osteoarthritis initiative. Front. Med..

[17] Mohajer B (2023). Levothyroxine use and longitudinal changes in thigh muscles in at-risk participants for knee osteoarthritis: Preliminary analysis from Osteoarthritis Initiative cohort. Arthritis Res. Ther..

[18] Rathbun AM (2018). Pain severity as a mediator of the association between depressive symptoms and physical performance in knee osteoarthritis. Osteoarthr. Cartil..

[19] Hunter DJ (2011). Evolution of semi-quantitative whole joint assessment of knee OA: MOAKS (MRI Osteoarthritis Knee Score). Osteoarthr. Cartil..

[20] Collins JE (2016). Semiquantitative imaging biomarkers of knee osteoarthritis progression: Data from the Foundation for the National Institutes of Health Osteoarthritis Biomarkers Consortium. Arthritis Rheumatol..

[21] Roemer FW (2015). What comes first? Multitissue involvement leading to radiographic osteoarthritis: Magnetic resonance imaging-based trajectory analysis over four years in the osteoarthritis initiative. Arthritis Rheumatol..

[22] Roemer FW (2015). Can structural joint damage measured with MR imaging be used to predict knee replacement in the following year?. Radiology.

[23] Xiong T (2023). Anserine bursa palpation tenderness is a risk factor for knee osteoarthritis progression and arthroplasty: Data from the Osteoarthritis Initiative. Clin. Rheumatol..

[24] Everhart JS (2020). Meniscus tears accelerate joint space loss and lateral meniscal extrusion increases risk of knee arthroplasty in middle-aged adults. J. Orthop. Res..

[25] Snoeker BAM (2021). Are structural abnormalities on knee MRI associated with osteophyte development? Data from the Osteoarthritis Initiative. Osteoarthr. Cartil..

[26] Costa CR, Morrison WB, Carrino JA (2004). Medial meniscus extrusion on knee MRI: Is extent associated with severity of degeneration or type of tear?. AJR Am. J. Roentgenol..

[27] Langhans MT, Lamba A, Saris DBF, Smith P, Krych AJ (2023). Meniscal extrusion: Diagnosis, etiology, and treatment options. Curr. Rev. Musculoskelet. Med..

[28] Roemer FW (2009). The association of meniscal damage with joint effusion in persons without radiographic osteoarthritis: The Framingham and MOST osteoarthritis studies. Osteoarthr. Cartil..

[29] Ramezanpour S (2023). Impact of sustained synovitis on knee joint structural degeneration: 4-year MRI data from the osteoarthritis initiative. J. Magn. Reson. Imaging.

[30] Hill CL (2007). Synovitis detected on magnetic resonance imaging and its relation to pain and cartilage loss in knee osteoarthritis. Ann. Rheum. Dis..

[31] Roemer FW (2022). Presence of magnetic resonance imaging-defined inflammation particularly in overweight and obese women increases risk of radiographic knee osteoarthritis: The POMA study. Arthritis Care Res. (Hoboken).

[32] Bacon K, LaValley MP, Jafarzadeh SR, Felson D (2020). Does cartilage loss cause pain in osteoarthritis and if so, how much?. Ann. Rheum. Dis..

[33] Kellgren JH, Lawrence JS (1957). Radiological assessment of osteo-arthrosis. Ann. Rheum. Dis..

[34] Lo GH (2022). Association between walking for exercise and symptomatic and structural progression in individuals with knee osteoarthritis: Data from the osteoarthritis initiative cohort. Arthritis Rheumatol..

[35] Bucci J (2022). Progression of knee osteoarthritis with use of intraarticular glucocorticoids versus hyaluronic acid. Arthritis Rheumatol..

[36] Roubille C (2015). Meniscal extrusion promotes knee osteoarthritis structural progression: Protective effect of strontium ranelate treatment in a phase III clinical trial. Arthritis Res. Ther..

[37] Adams JG, McAlindon T, Dimasi M, Carey J, Eustace S (1999). Contribution of meniscal extrusion and cartilage loss to joint space narrowing in osteoarthritis. Clin. Radiol..

[38] Hellio Le Graverand MP, Vignon E, Otterness IG, Hart DA (2001). Early changes in lapine menisci during osteoarthritis development: Part I: Cellular and matrix alterations. Osteoarthr. Cartil..

[39] Roth M (2018). Sensitivity to change and association of three-dimensional meniscal measures with radiographic joint space width loss in rapid clinical progression of knee osteoarthritis. Eur. Radiol..

[40] Buckland-Wright JC, Macfarlane DG, Lynch JA, Jasani MK, Bradshaw CR (1995). Joint space width measures cartilage thickness in osteoarthritis of the knee: High resolution plain film and double contrast macroradiographic investigation. Ann. Rheum. Dis..

[41] Driban JB (2019). Accelerated knee osteoarthritis is characterized by destabilizing meniscal tears and preradiographic structural disease burden. Arthritis Rheumatol..

[42] Englund M (2009). Meniscal tear in knees without surgery and the development of radiographic osteoarthritis among middle-aged and elderly persons: The Multicenter Osteoarthritis Study. Arthritis Rheum..

[43] Lee H (2021). A Guideline for reporting mediation analyses of randomized trials and observational studies: The AGReMA statement. JAMA.

[44] Scanzello CR, Goldring SR (2012). The role of synovitis in osteoarthritis pathogenesis. Bone.

[45] Belluzzi E (2019). Contribution of infrapatellar fat pad and synovial membrane to knee osteoarthritis pain. Biomed Res. Int..

[46] Xie J, Huang Z, Yu X, Zhou L, Pei F (2019). Clinical implications of macrophage dysfunction in the development of osteoarthritis of the knee. Cytokine Growth Factor Rev..

[47] Miller TT, Staron RB, Feldman F, Cepel E (1997). Meniscal position on routine MR imaging of the knee. Skelet. Radiol..

[48] Breitenseher MJ (1997). MR imaging of meniscal subluxation in the knee. Acta Radiol..

[49] Atkinson HF (2021). Association between changes in knee load and effusion-synovitis: Evidence of mechano-inflammation in knee osteoarthritis using high tibial osteotomy as a model. Osteoarthr. Cartil..

[50] Hart HF (2018). Relation of meniscus pathology to prevalence and worsening of patellofemoral joint osteoarthritis: The Multicenter Osteoarthritis Study. Osteoarthr. Cartil..

[51] Antony B (2017). The relationship between meniscal pathology and osteoarthritis depends on the type of meniscal damage visible on magnetic resonance images: Data from the Osteoarthritis Initiative. Osteoarthr. Cartil..

[52] Scanzello CR (2011). Synovial inflammation in patients undergoing arthroscopic meniscectomy: Molecular characterization and relationship to symptoms. Arthritis Rheum..

[53] Loeuille D (2005). Macroscopic and microscopic features of synovial membrane inflammation in the osteoarthritic knee: Correlating magnetic resonance imaging findings with disease severity. Arthritis Rheum..

