# Supplementary material for: The mediating role of synovitis in meniscus pathology and knee osteoarthritis radiographic progression
Source: Sci Rep. 2024 May 29;14:12335. doi: 10.1038/s41598-024-63291-6 (PMC11137050; doi:10.1038/s41598-024-63291-6)
Supplement: Supplementary file 1 — Supplementary Table 1. [file 41598_2024_63291_MOESM1_ESM.docx]

**Supplementary Table 1. Analysis of the association between medial meniscus pathology and radiology progression mediated by the total synovitis score.**

|  | KL progression^a^ | | |  | | Medial JSN progression^a^ | | | | | |
| --- | --- | --- | --- | --- | --- | --- | --- | --- | --- | --- | --- |
|  | *β* | 95%CI | *P*-value |  | | *β* | | 95%CI | | *P*-value | |
| ALL^b^ | | | | | | | | | | | |
| Baseline | | | | | | | | | | | |
| Indirect effect | 0.020 | 0.003, 0.043 | 0.07 |  | 0.017 | | 0.002, 0.041 | | 0.10 | |  |
| Direct effect | 0.167 | 0.062, 0.266 | **<0.01** |  | 0.195 | | 0.094, 0.303 | | **<0.01** | |  |
| Total effect | 0.187 | 0.080, 0.290 | **<0.01** |  | 0.212 | | 0.107, 0.321 | | **<0.01** | |  |
| Mediation |  |  |  |  |  | |  | |  | |  |
| Baseline to 24 months | | | | | | | | | | |  |
| Indirect effect | 0.031 | 0.009, 0.060 | **0.02** |  | 0.038 | | 0.010, 0.072 | | **0.02** | |  |
| Direct effect | 0.155 | 0.053, 0.257 | **<0.01** |  | 0.177 | | 0.075, 0.280 | | **<0.01** | |  |
| Total effect | 0.186 | 0.078, 0.289 | **<0.01** |  | 0.214 | | 0.109, 0.323 | | **<0.01** | |  |
| Mediation | 16.7% |  |  |  | 17.8% | |  | |  | |  |

^a^Adjusted for sex, age, race, body mass index, varus alignment, respective compartment meniscal posterior root tear, injury, surgery and Kellgren and Lawrence (KL) grade at baseline.

^b^There were both meniscal extrusion and meniscal damage (tear or maceration).
